# Supplementary material for: PCLAF-DREAM drives alveolar cell plasticity for lung regeneration
Source: Nat Commun. 2024 Oct 24;15:9169. doi: 10.1038/s41467-024-53330-1 (PMC11502753; doi:10.1038/s41467-024-53330-1)
Supplement: Supplementary file 1 — Supplementary Information [file 41467_2024_53330_MOESM1_ESM.pdf]

List of Supplementary Materials

Supplementary Figures 1 to 20

Supplementary Figures

Supplementary Fig. 1. Cell type annotation of human and mouse lung single-cell transcriptomics
Supplementary Fig. 2. Experimental scheme for generating *Pclaf-LacZ* mice
Supplementary Fig. 3. *Pclaf* KO inhibits AT1 regeneration and promotes AT2 repopulation.
Supplementary Fig. 4. Lung organoid culture system
Supplementary Fig. 5. *Pclaf* KO inhibits alveolar-type lung organoid formation.
Supplementary Fig. 6. Scheme of single-cell transcriptomics of *Pclaf* WT and KO mouse lung tissues
Supplementary Fig. 7. Cell type annotation of mouse lung single-cell transcriptomics
Supplementary Fig. 8. *Pclaf* KO-impaired cell lineage trajectory from AT2 cells to AT1 cells
Supplementary Fig. 9. PAPCs express various lung epithelial cell markers.
Supplementary Fig. 10. Both germline and conditional KO of *Pclaf* inhibit AT1 cell generation from AT2 cells
Supplementary Fig. 11. *Pclaf* KO downregulates DREAM-target gene expression in PAPC.
Supplementary Fig. 12. Transduction efficiency of lentiviruses encoding Lin52-WT or Lin52 S28A in LOs
Supplementary Fig. 13. Impact of *Pclaf* KO on Wnt signaling, Sox9, and Myc transcriptional signatures
Supplementary Fig. 14. *Clic4* downregulation is associated with decreased activity of TGF-β signaling in *Pclaf* KO PAPCs.
Supplementary Fig. 15. Pharmacological or genetic activation of the DREAM axis rescues *Pclaf* KO-inhibited TGF-β signaling.
Supplementary Fig. 16. TGF-β rescues *Pclaf* KO-impaired cell plasticity.
Supplementary Fig. 17. Elevated TGF-β signaling in inflamed lesions of *Pclaf* KO lung tissues
Supplementary Fig. 18. Elevated TGF-β signaling in lung fibroblasts of IPF patients
Supplementary Fig. 19. Phenelzine promotes LO formation.
Supplementary Fig. 20. Source data

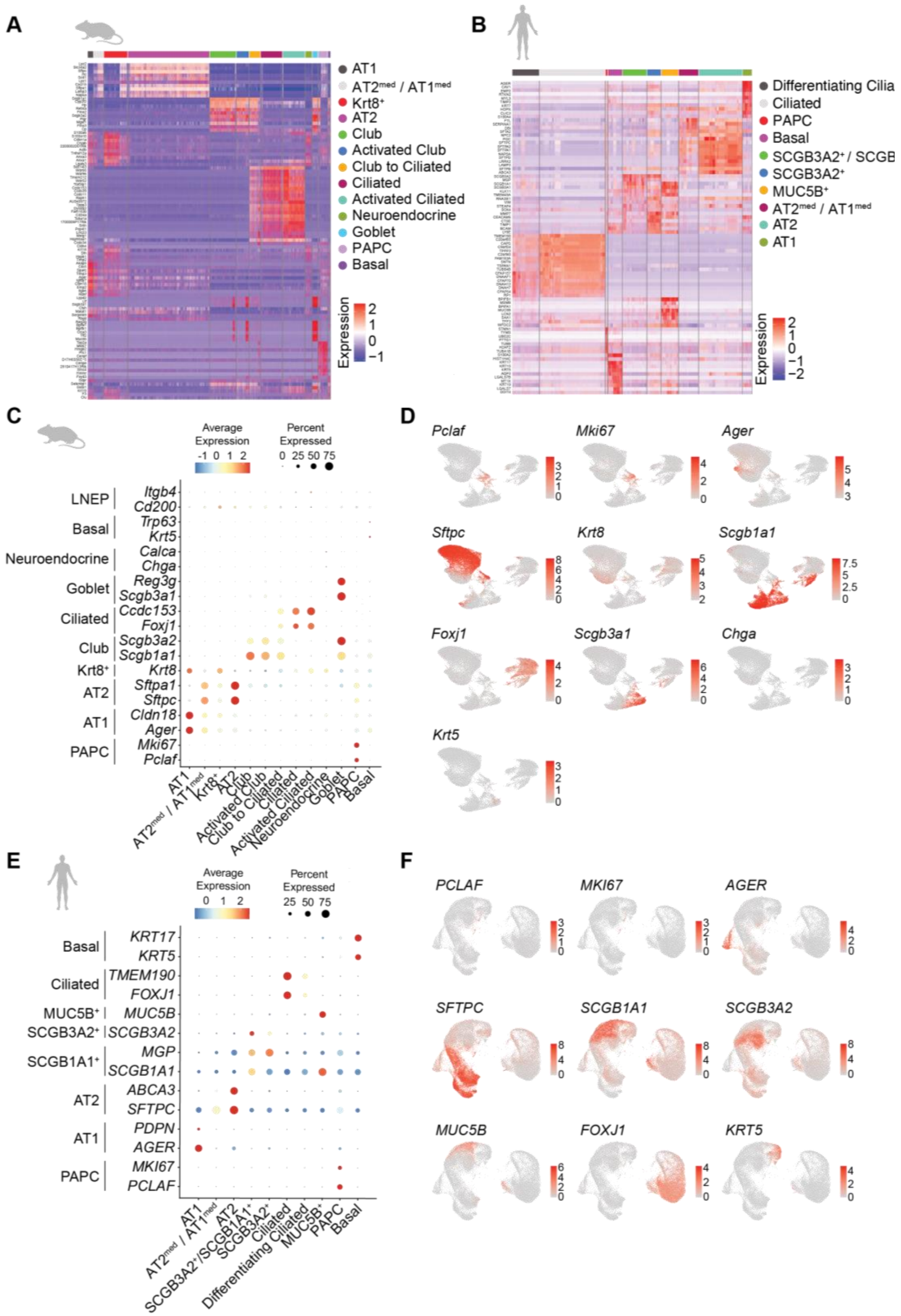

Supplementary Fig. 1. Cell type annotation of human and mouse lung single-cell transcriptomics
A Heatmap of gene expression of the top 10 genes in each cell type of mouse lung using data shown in Figure 1A. **B** Heatmap of gene expression of the top 10 genes in each cell type of human lung using data shown in Figure 1D. **C** Dot plots for mouse lung epithelial marker gene expression in each cell type. **D** Feature plots of mouse lung epithelial marker gene expression. **E** Dot plots for human lung epithelial marker gene expression in each cell type. **F** Feature plots of human lung epithelial marker gene expression. Graphic icons were created with BioRender.com.

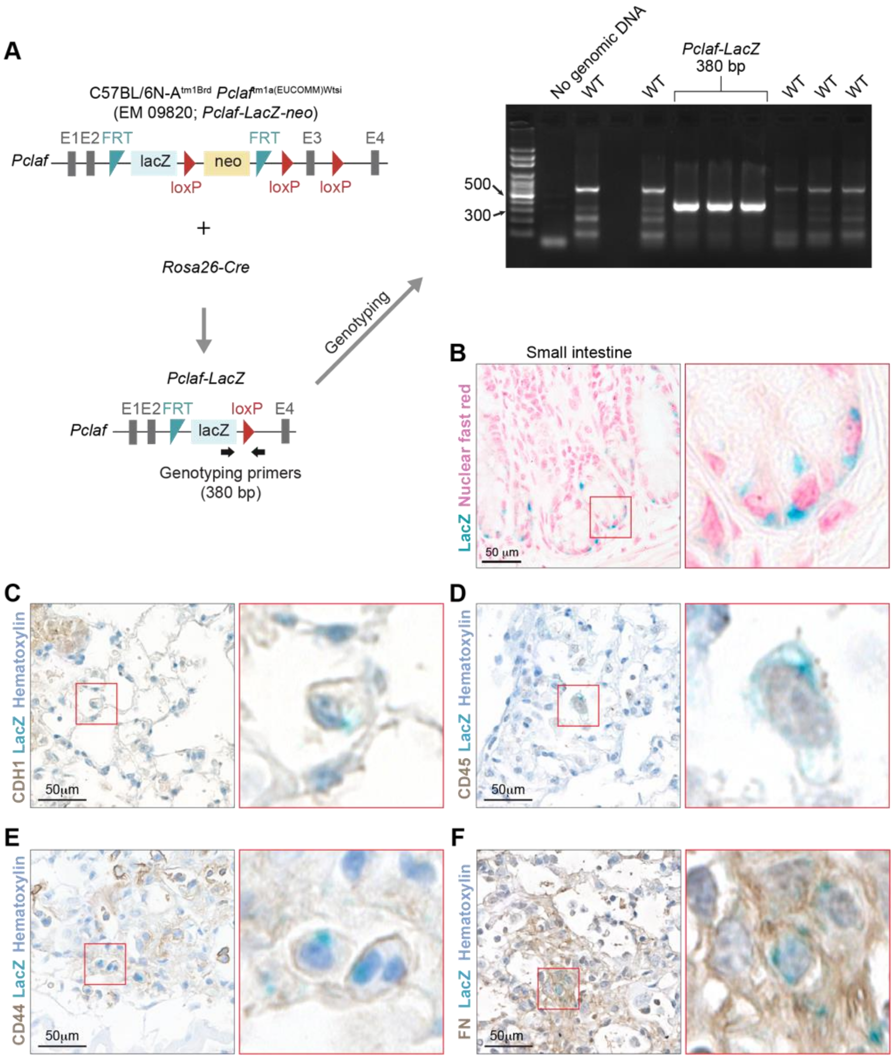

Supplementary Fig. 2. Experimental scheme for generating *Pclaf-LacZ* mice

A Experimental scheme for generating *Pclaf-LacZ* mice. C57BL/6N-A^{tm1Brd} *Pclaf*$^{tm1a(EUCOMM)Wtsi}$/WtsiPh mice were bred with the *Rosa26-Cre* strain. The *Pclaf-LacZ* allele was confirmed by PCR-based genotyping. **B** Representative image of X-gal staining of the small intestine where PCLAF is specifically expressed in the crypt [1]. PCLAF-LacZ was expressed in the crypt base columnar cells. **C-F** *Pclaf-LacZ* mice were treated with bleomycin (1.4 U/kg, by intratracheal instillation). At 7 dpi, lungs were collected and processed for immunostaining: epithelial cell (**C**; CDH1), immune cell (**D**; CD45), or mesenchymal cell (**E**; CD44 and **F**; fibronectin (FN)) in combination with X-gal staining. Represented images are shown (n≥3). Panel A was created with BioRender.com.

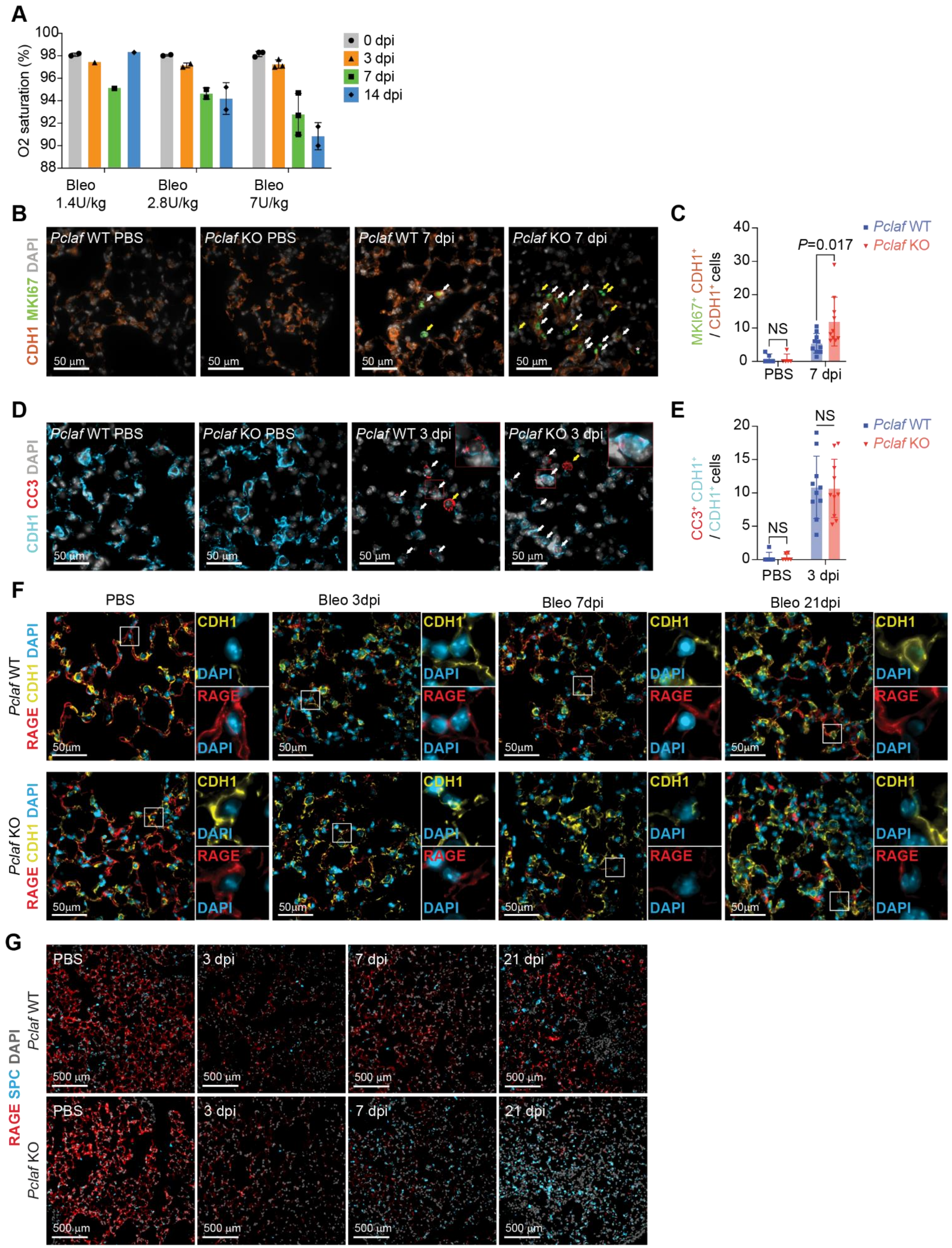

Supplementary Fig. 3. *Pclaf* KO inhibits AT1 regeneration and promotes AT2 repopulation.

A 8-week-old C57BL/6 mice were treated with bleomycin (1.4 U/kg; 0 dpi [n=2], 3, 7, 21 dpi [each n=1], 2.8 U/kg; n=2, 7.0 U/kg; n=3; intratracheal instillation). The dynamics of SpO_2 levels were measured by pulse-oximetry at the indicated time points. One mouse instilled with bleomycin (1.4 U/kg) was dead at 1 dpi. Mouse instilled with 1.4 U/kg bleomycin showed recovered SpO_2 level back to levels of 0 dpi at 14 dpi, while mice with 2.8 U/kg or 7 U/kg of bleomycin showed inhibited SpO_2 level at 14 dpi. **B-G** Experimental scheme for the bleomycin-induced lung injury model. *Pclaf* WT and KO mice were treated with phosphate-buffered saline (PBS) (n=5 for *Pclaf* WT, n=5 for *Pclaf* KO) or bleomycin (1.4 U/kg; n=10 for *Pclaf* WT, n=10 for *Pclaf* KO) by intratracheal instillation. Representative images of immunostaining for CDH1 and MKI67 at indicated time points (**B**). Quantification graph of $CDH1^+/MKI67^+$ cells / $CDH1^+$ cells (**C**). Representative images of immunostaining for CDH1 and cleaved caspase 3 (CC3) at indicated time points (**D**). Quantification graph of $CDH1^+/CC3^+$ cells / $CDH1^+$ cells (**E**). Representative images of immunostaining for RAGE and CDH1 at indicated time points (**F**). Representative images of immunostaining for RAGE and SPC at indicated time points (**G**). Represented images are shown ($n \geq 3$).

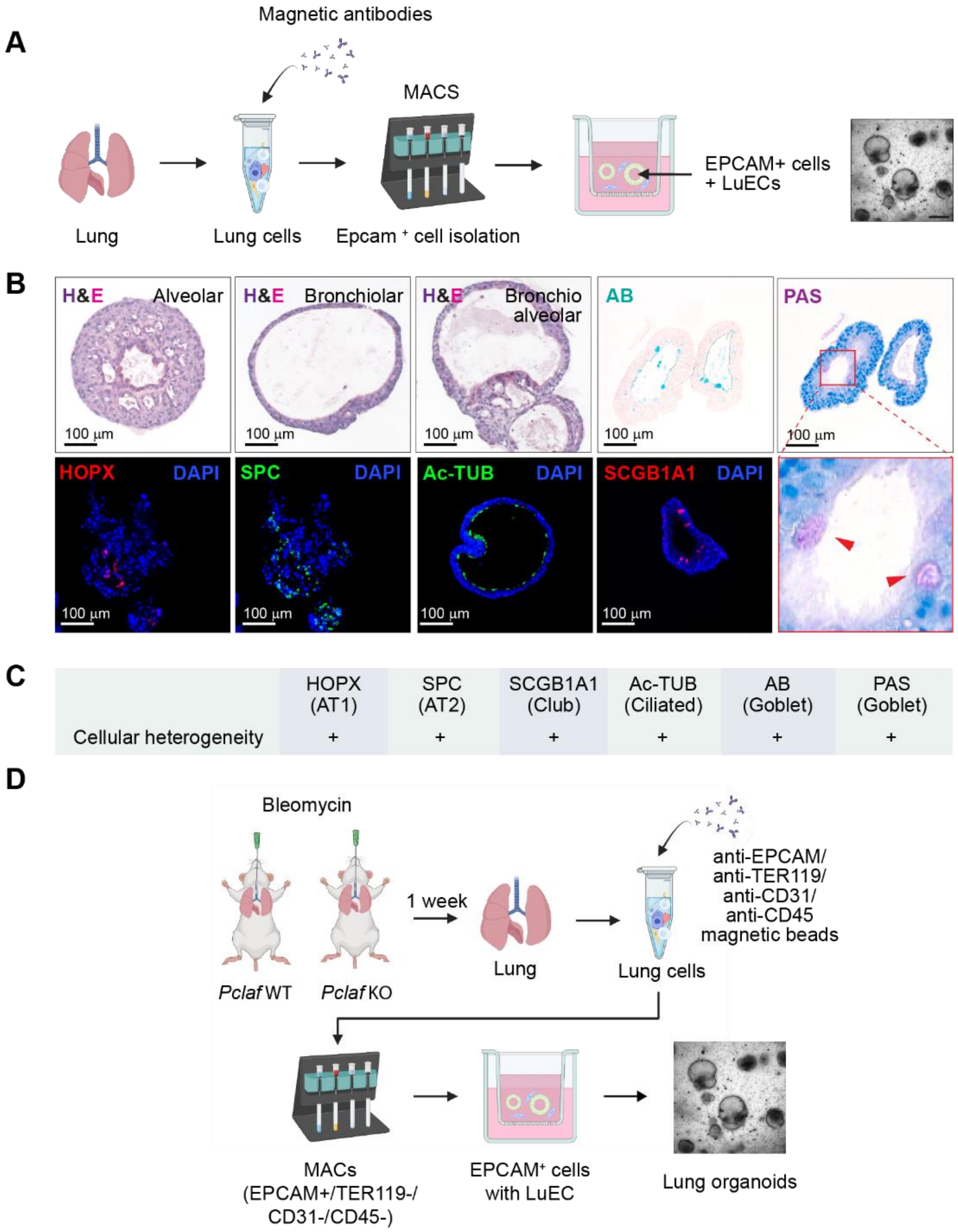

Supplementary Fig. 4. Lung organoid culture system

A Scheme of LO culture. The lung epithelial cells were isolated from WT mice by magnetic-activated cell sorting (MACS) and cultured with LuECs at a liquid-air interface to grow LOs. **B** Representative images of organoids for hematoxylin and eosin (H&E), alcian blue (AB), and periodic acid-Schiff (PAS) staining (*upper* panels), and immunostained for HOPX (AT1 cells), SPC (AT2 cells), acetyl-Tubulin (Ac-TUB; Ciliated cells), and SCGB1A1 (Club cells) of organoids with LuECs. **C** Table of positive cells using data shown in Supplementary Fig 4B. **D** Scheme of LO culture. The lung epithelial cells were isolated from bleomycin-treated lungs of *Pclaf* WT or *Pclaf* KO mice at 7 dpi by MACS. The lung epithelial cells ($TER119^-/CD31^-/CD45^-/EPCAM^+$) were cultured with lung endothelial cells ($CD31^+$) at a liquid-air interface to generate LOs. Represented images are shown (n≥3). Panel A and D were created with BioRender.com.

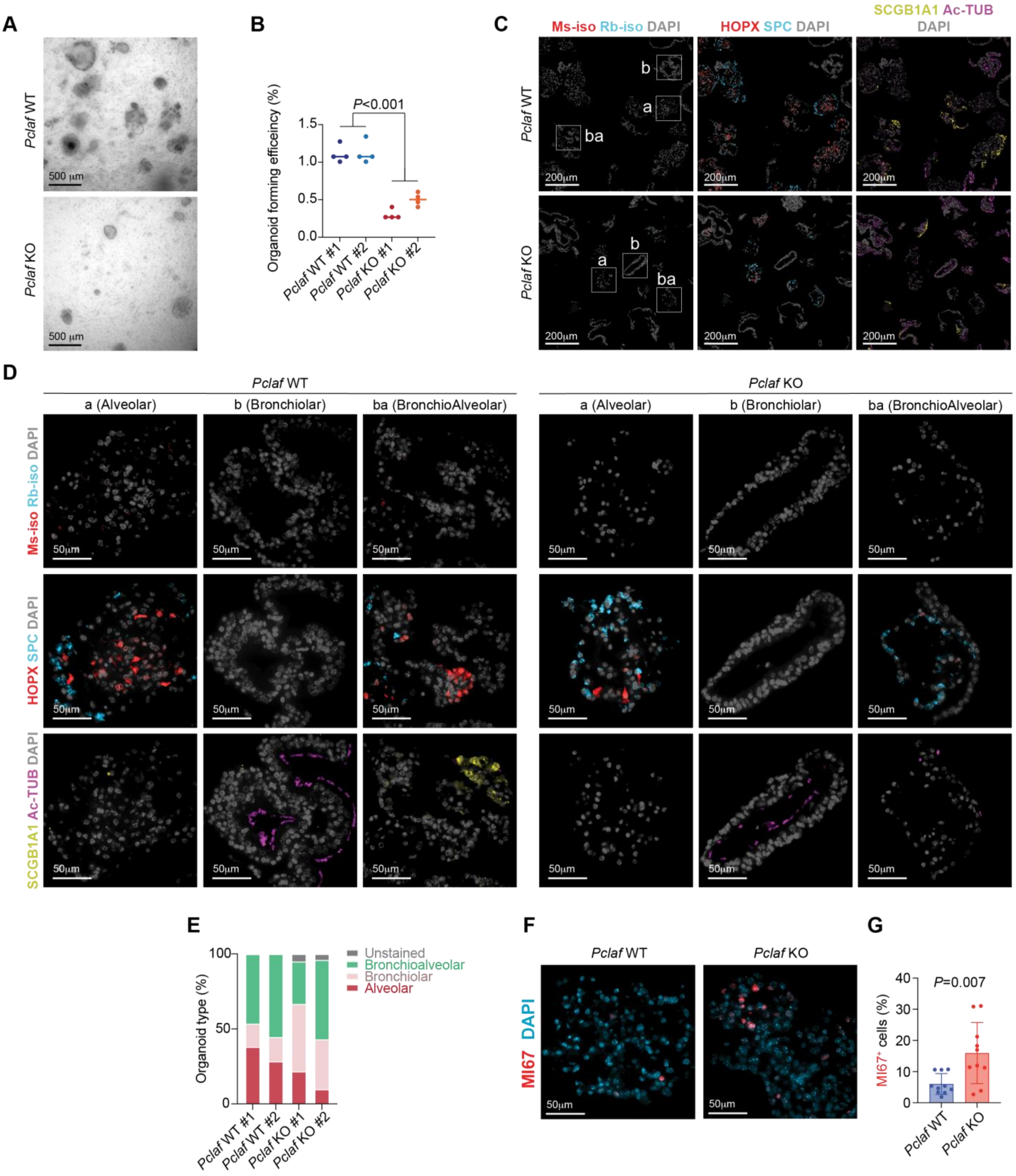

Supplementary Fig. 5. *Pclaf* KO inhibits alveolar-type lung organoid formation.
The lung epithelial cells were isolated from bleomycin-treated lungs of *Pclaf* WT or *Pclaf* KO mice at 7 dpi (bleomycin administration) by magnetic-activated cell sorting (MACS). The lung epithelial cells (TER119$^-$/CD31$^-$/CD45$^-$/EPCAM$^+$) were cultured with lung endothelial cells (CD31$^+$) at a liquid-air interface to generate LOs. **A** Bright-field z-stack images of LOs at day 12 (n=4). **B** Quantification graph of lung organoid forming efficiency (OFE). **C** Representative images of LOs that were immunostained for RAGE (AT1 cells), SPC (AT2 cells) Ac-TUB (Ciliated cells), and SCGB1A1 (Club cells). Isotype-controls (rabbit IgG for SPC and SCGB1A1, mouse IgG

for HOPX and Ac-TUB) served as negative controls. Serial sections were used for isotype-control, HOPX and SPC, and Ac-TUB and SCGB1A1 staining. a; alveolar, b; bronchiolar, ba; bronchioalveolar. **D** High magnification images of each alveolar, bronchiolar, and bronchioalveolar organoids shown and indicated in Supplementary Figure 5C. **E** Quantification graph of organoid type. #1 and #2 indicate two independent experiment sets. **F** Representative images of LOs that were immunostained for MKI67 (n=10). **G** Quantification graph of $MKI67^+$ cells / $DAPI^+$ cells. Two-tailed Student's *t*-test; error bars: SD. Represented images and data are shown ($n \geq 3$).

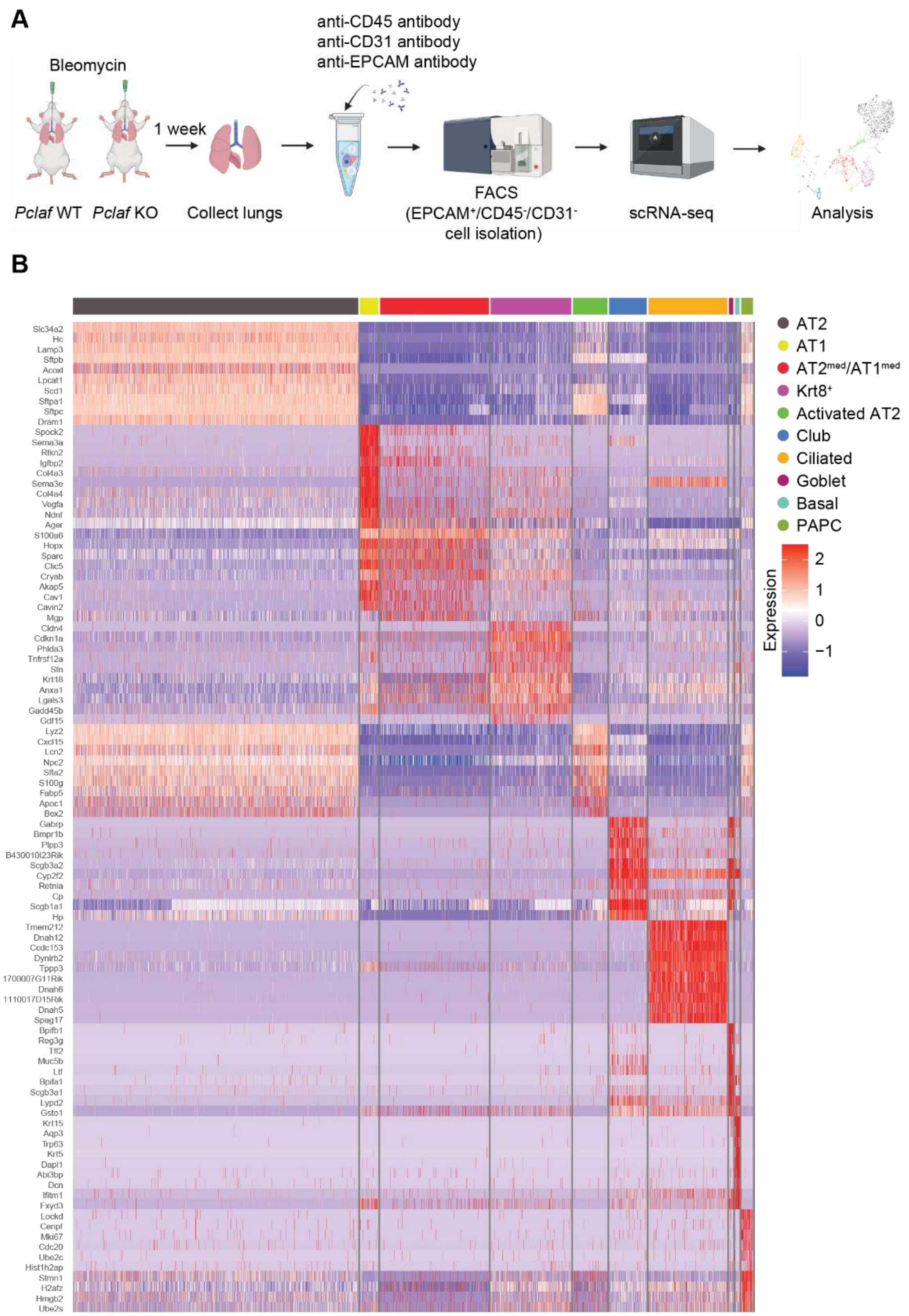

Supplementary Fig. 6. Scheme of single-cell transcriptomics of *Pclaf* WT and KO mouse lung tissues

A Scheme of scRNA-seq. Lung epithelial cells were isolated from *Pclaf* WT and KO mice (treated with bleomycin) by fluorescence-activated cell sorting (FACS). $CD31^-/CD45^-/EPCAM^+$ cells were used to generate sequencing data using the 10X Genomics single-cell sequencing platform. **B** Heatmap of gene expression-based cell clusters of the top 10 genes in each cell type of mouse lung using data shown in Figure 3B. Panel A was created with BioRender.com.

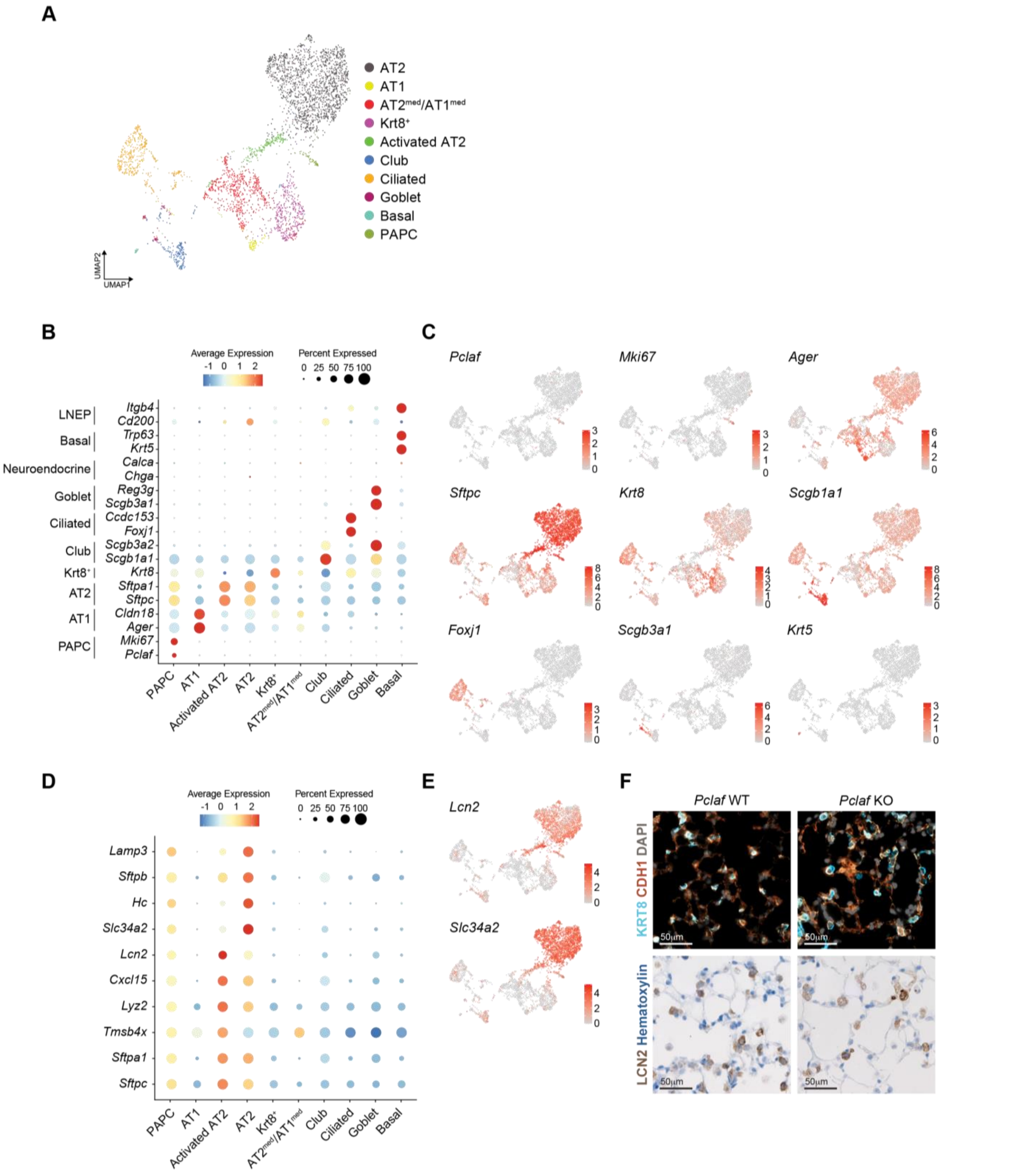

Supplementary Fig. 7. Cell type annotation of mouse lung single-cell transcriptomics

Analysis of the scRNA-seq dataset is shown in Figure 3B. **A** UMAP displays each cell cluster, colored by cell type. **B** Dot plots for mouse lung epithelial marker gene expression quantification of each cell type. **C** Feature plots of mouse lung epithelial marker gene expression. **D** Dot plots for genes specifically expressed in AT2 and activated AT2 cells. **E** Feature plots displaying the expression of *Lcn2* and *Slc34a2*. **F** Representative images of immunostaining for KRT8 and CDH1, or LCN2 using lung slides at 7 dpi as shown in Figure 2. Represented images and data are shown (n≥3).

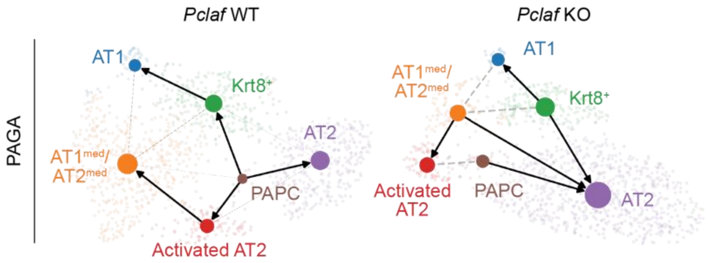

Supplementary Fig. 8. *Pclaf* KO-impaired cell lineage trajectory from AT2 cells to AT1 cells
Partition-based graph abstraction (PAGA) analysis using the results of RNA velocity analysis shown in Figure 3C.

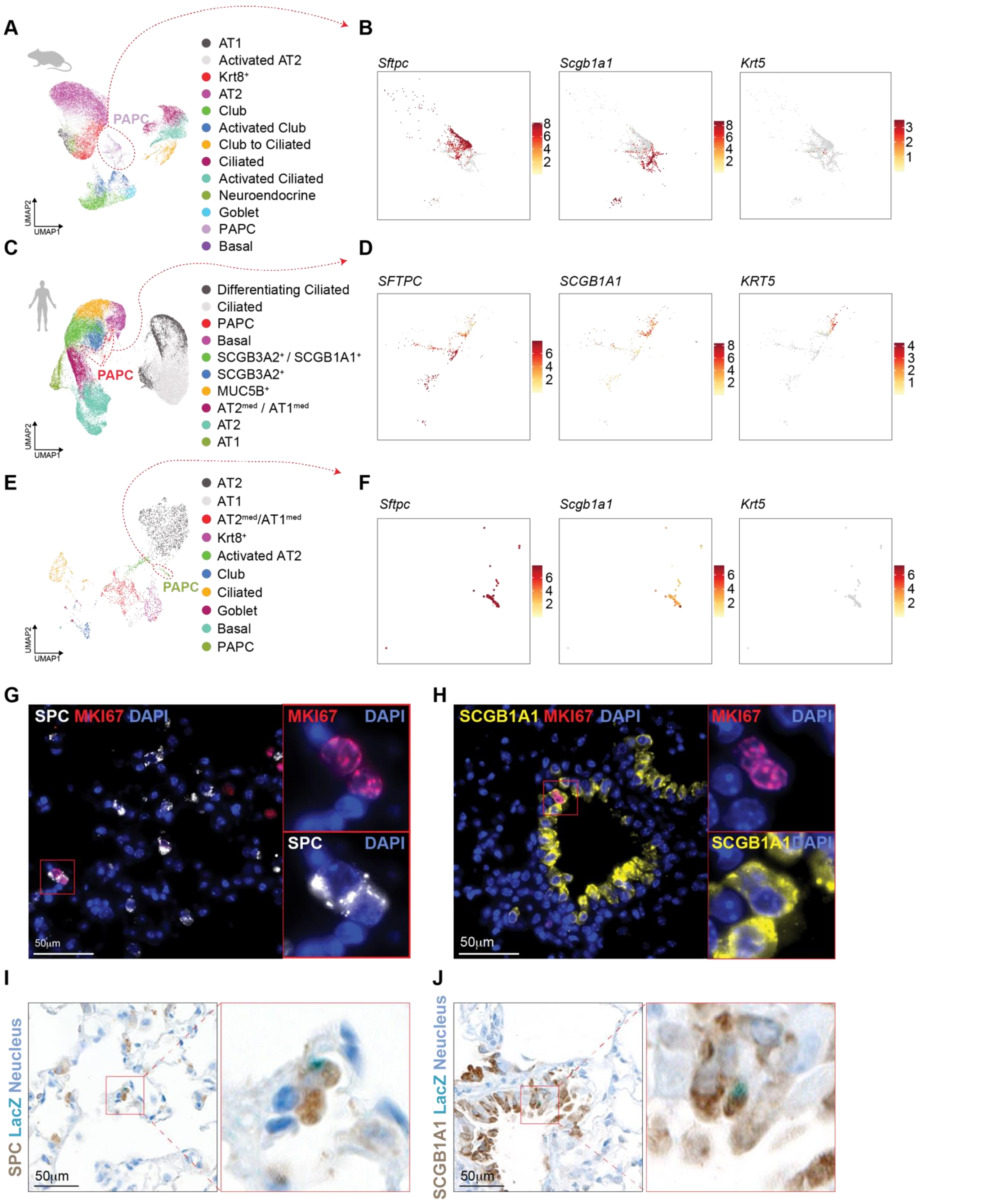

Supplementary Fig. 9. PAPCs express various lung epithelial cell markers.

A UMAPs displaying each cell cluster, colored by cell types of mouse lung shown in Figure 1A. **B** Feature plots displaying the expression of *Sftpc, Scgb1a1,* and *Krt5* in mouse PAPC cells. **C** UMAPs displaying each cell cluster, colored by cell types of human lung shown in Figure 1D. **D** Feature plots displaying the expression of *SFTPC, SCGB1A1,* and *KRT5* in human PAPC cells. **E** UMAPs displaying each cell cluster, colored by cell

types of mouse lung (*Pclaf* WT and KO at 7 dpi) shown in Figure 3B. **F** Feature plots displaying the expression of *Sftpc, Scgb1a1,* and *Krt5* in mouse PAPC cells. **G** Representative images of immunostaining for SPC and MKI67 using lung slides at 7 dpi shown in Figure 2. **H** Representative images of immunostaining for SCGB1A1 and MKI67 using lung slides at 7 dpi shown in Figure 2. **I, J** Representative images of immunostaining for SPC (**I**) and SCGB1A1 (**J**) in combination with X-gal staining, using lung slides at 7 dpi are shown in Supplementary Figure 2. Representative images are shown (n≥3). Graphic icons were created with BioRender.com.

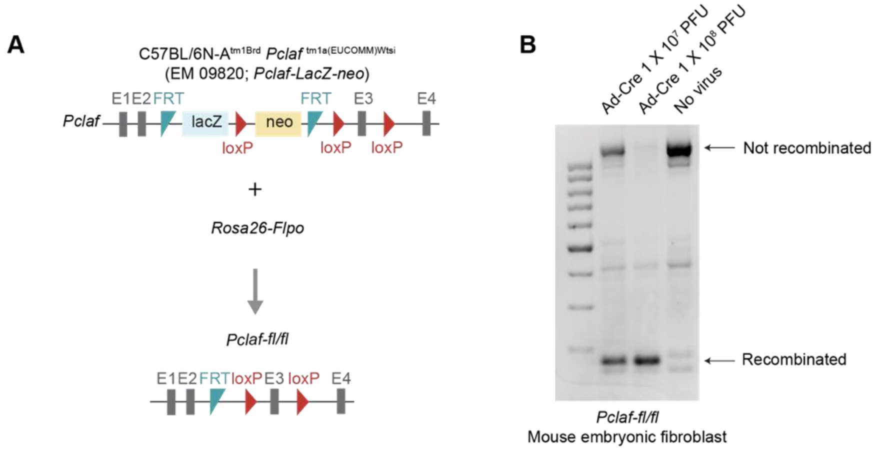

Supplementary Fig. 10. Both germline and conditional KO of *Pclaf* inhibit AT1 cell generation from AT2 cells

A Experimental scheme of generating of *Pclaf-fl/fl* mice. **B** Mouse embryonic fibroblasts (MEFs) were isolated from *Pclaf-fl (floxed)/fl* mice. *Pclaf-fl/fl* MEFs were treated with Ad-Cre viruses (1 $\times$ 10^7 or 1 $\times$ 10^8 PFU). Conditional knock-out (cKO) by Ad-Cre was checked by PCR-based genotyping. FIgure A was created with BioRender.com.

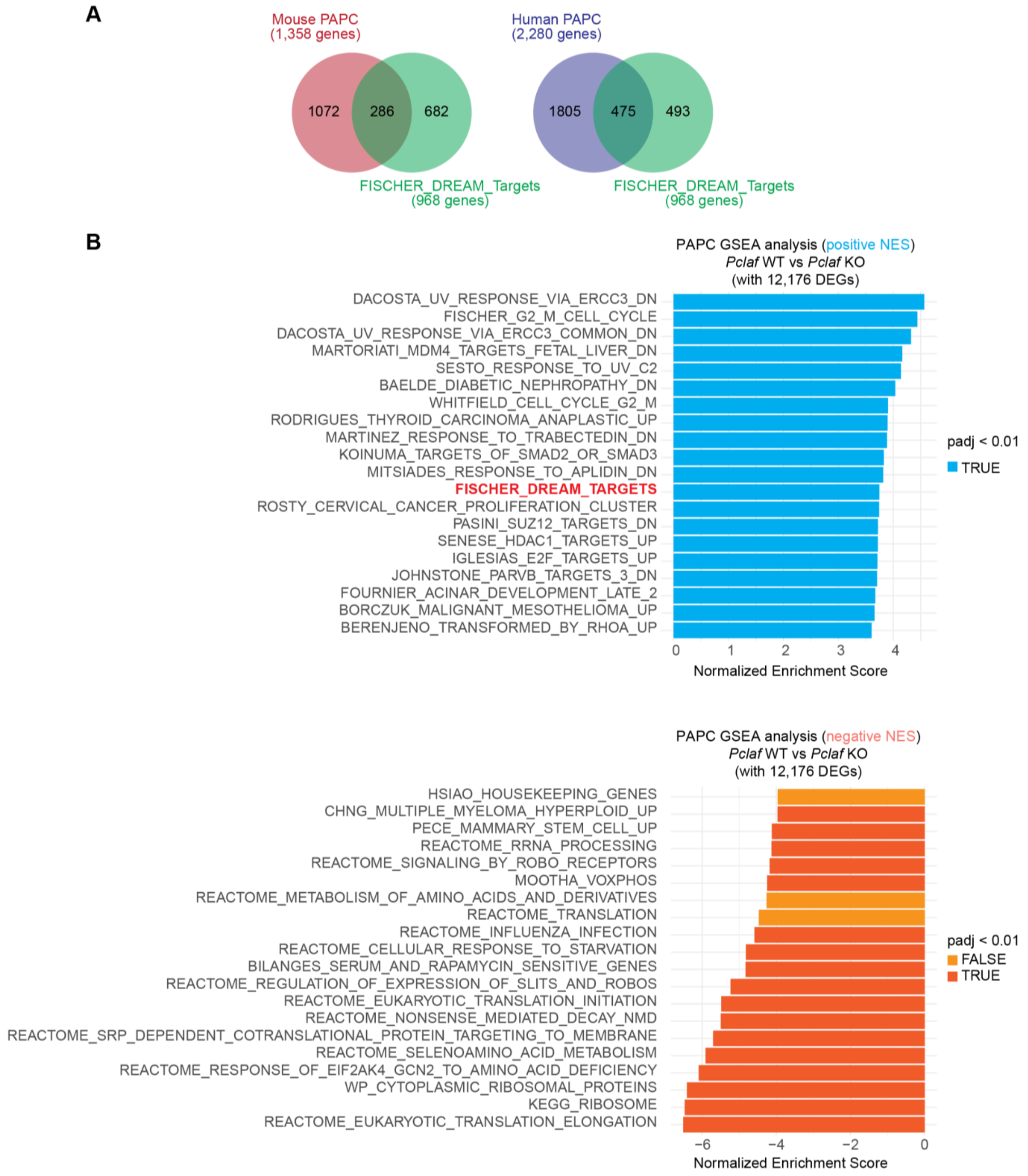

Supplementary Fig. 11. *Pclaf* KO downregulates DREAM-target gene expression in PAPC.
A Venn diagram analysis of DREAM target genes *vs.* genes specifically expressed in the PAPC clusters (mouse scRNA-seq dataset shown in Figure 1A or the human scRNA-seq dataset shown in Figure 1D). **B** Top 20 gene sets (*upper* panel) and bottom 20 gene sets (*bottom* panel) of GSEA with 12,176 DEGs between *Pclaf* WT PAPCs and *Pclaf* KO PAPCs in the scRNA-seq dataset shown in Figure 3.

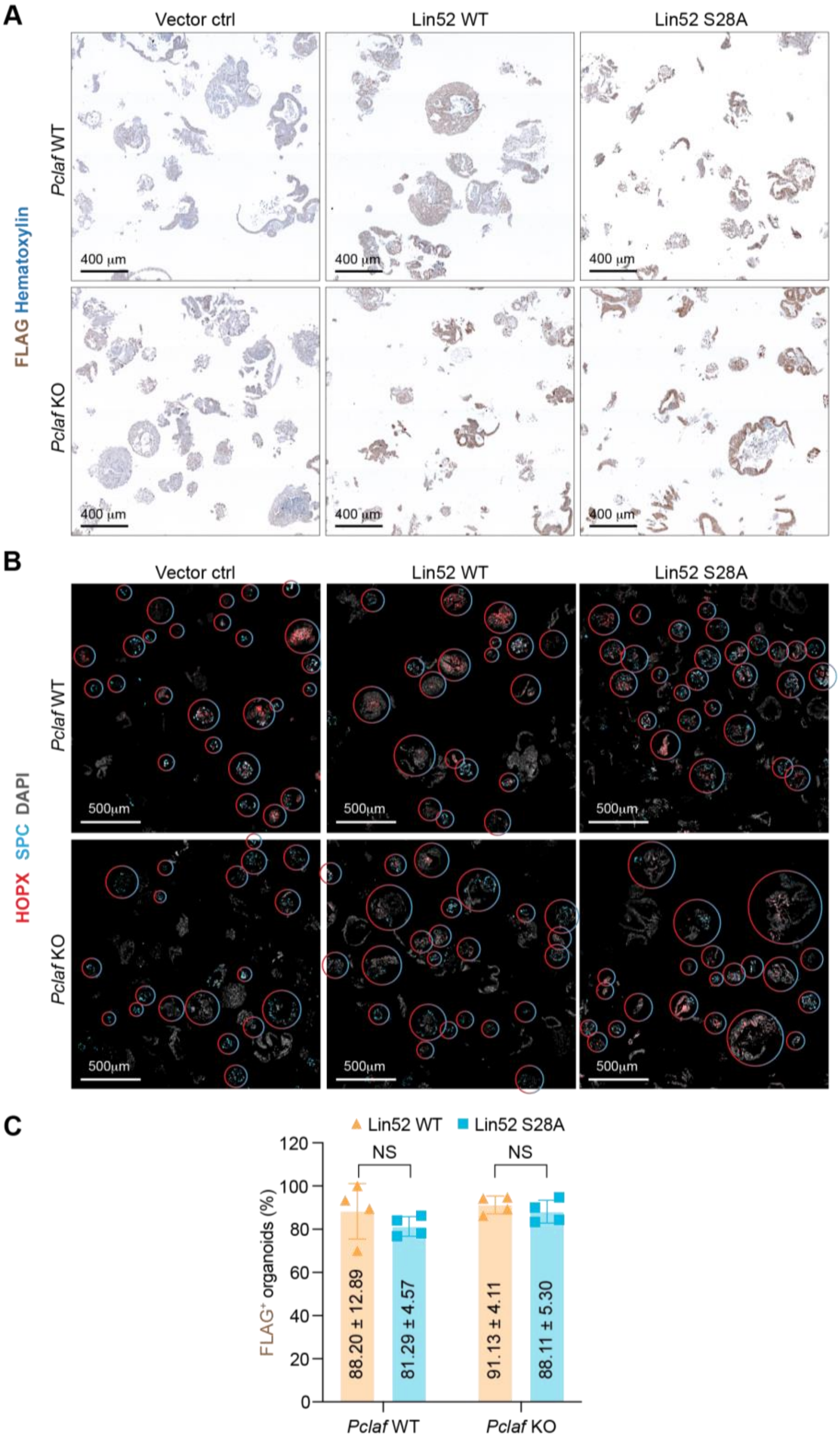

Supplementary Fig. 12. Transduction efficiency of lentiviruses encoding Lin52-WT or Lin52 S28A in LOs Isolated lung epithelial cells were transduced with RFP, Lin52 WT, or Lin52 S28A by lentivirus and then cultured with LO. **A** Representative images of chemically immunostained for FLAG. **B** Representative images of immunofluorescent (IF) staining for HOPX (AT1) and SPC (AT2) on day 14. The circles (in red-light blue mixed) indicate the relative ratio of $HOPX^+$ to SPC^+ cells in LOs. **C** Quantification of $FLAG^+$ LOs (n=4). Two-tailed Student's *t*-test; error bars: SD. Representative images are shown ($n \geq 3$).

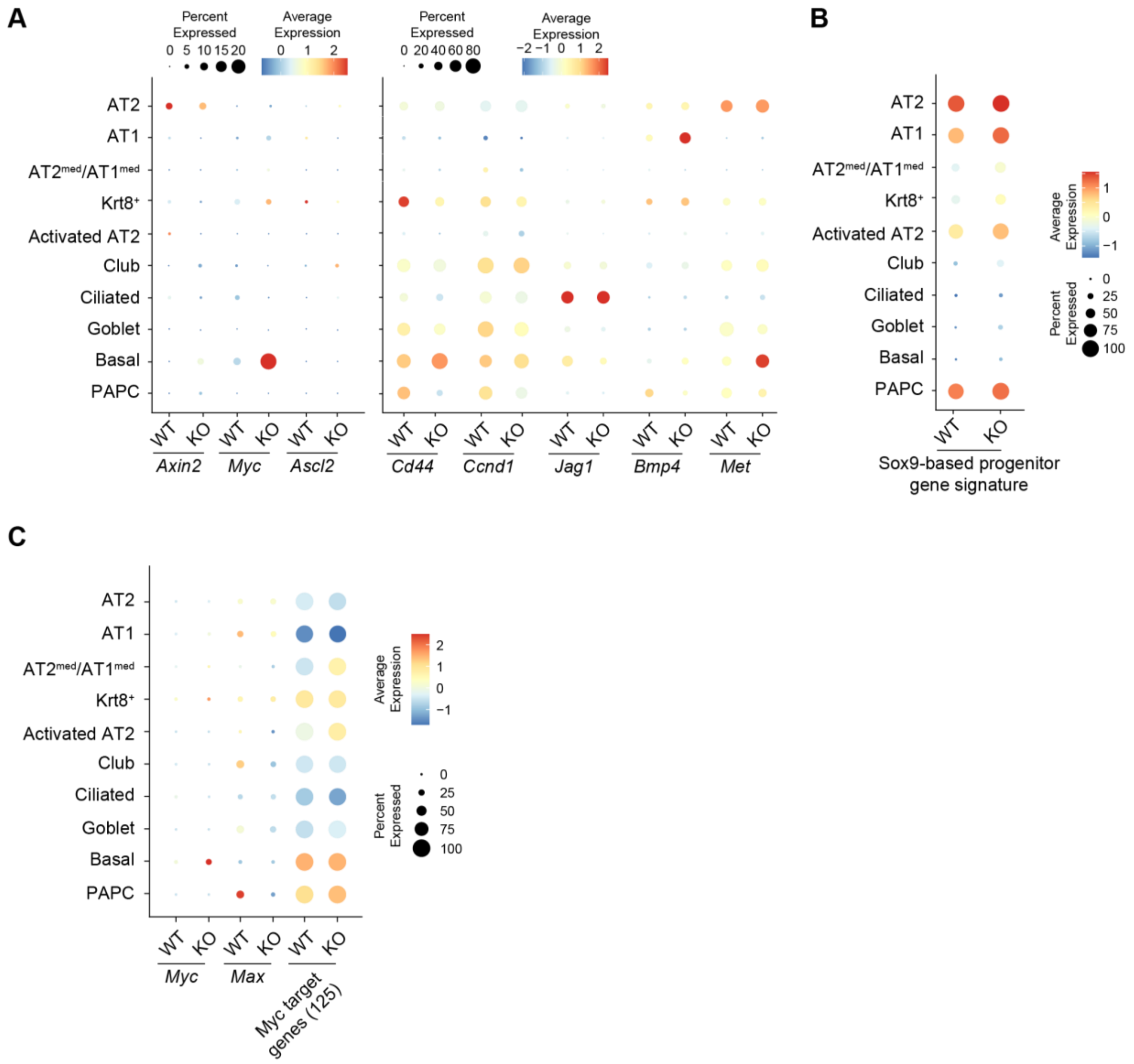

Supplementary Fig. 13. Impact of *Pclaf* KO on Wnt signaling, Sox9, and Myc transcriptional signatures
A-**C** Dot plots displaying each gene expression from the scRNA-seq dataset shown in Figure 3. Dot plots showing the expression of Wnt signaling target genes in each cell type (**A**). Dot plots depicting transcriptional module scores of the Sox9-based progenitor cell signature gene set (**B**). Dot plots showing the expression of *Myc* and *Max* and transcriptional module scores of the Myc target gene set (**C**). *Pclaf* KO barely alters Wnt signaling and Sox9-based progenitor transcriptional signature.

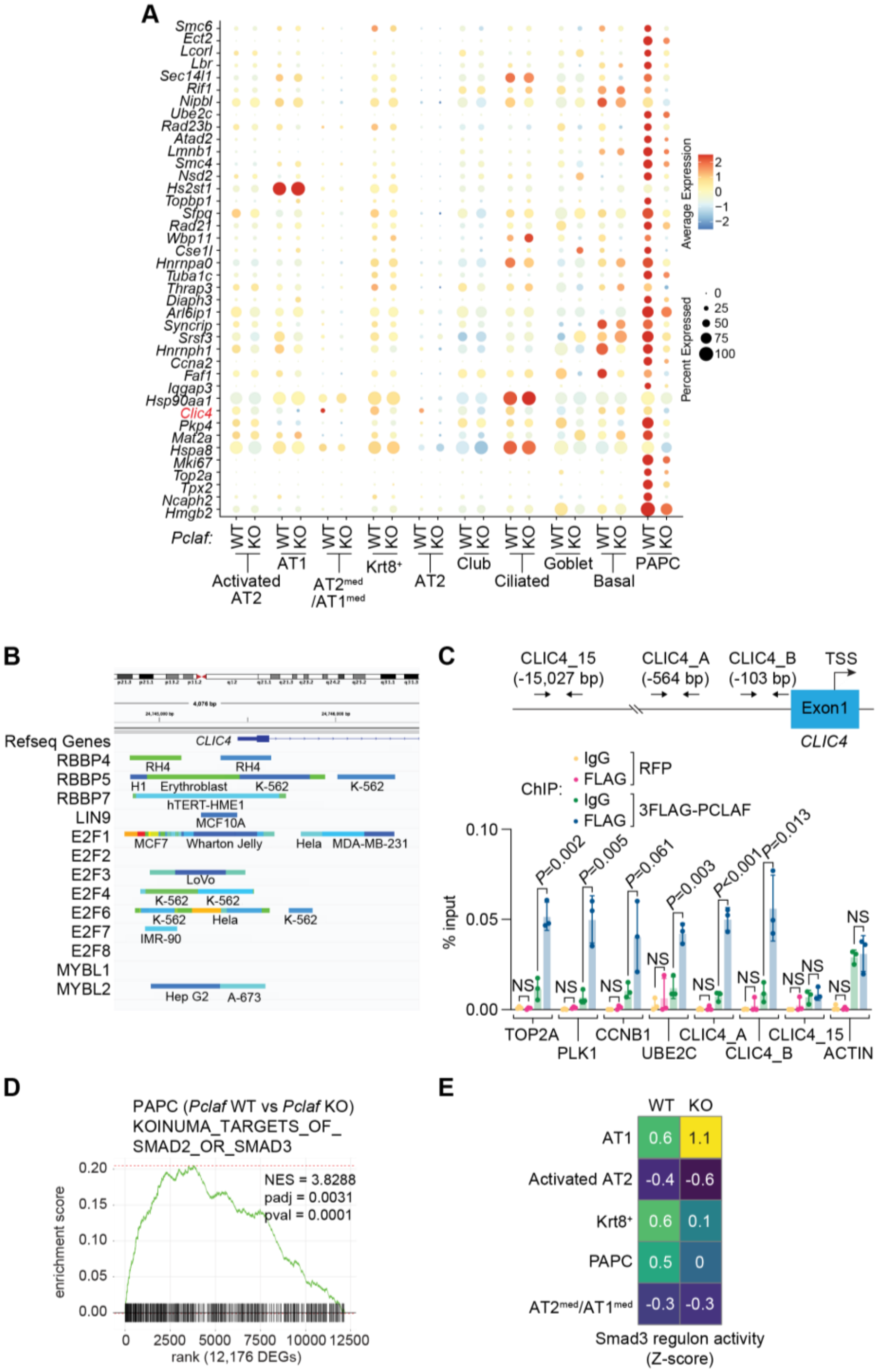

Supplementary Fig. 14. *Clic4* downregulation is associated with decreased activity of TGF-β signaling in *Pclaf* KO PAPCs.

A Dot plots displaying the top 40 differently expressed DREAM target genes in each cell type. **B** The DREAM complex components bind to the *CLIC4* promoter in ChIP-Atlas database (chip-atlas.org). **C** qPCR analysis using indicated primer sets targeting proximal promoter of DREAM target genes, *CLIC4*, or *ACTB*. ChIP was performed using anti-FLAG antibody. H358 cells ectopically expressing 3FLAG-*PCLAF* were used for ChIP (n=3). **D** GSEA of *Pclaf* WT vs. *Pclaf* KO in the PAPC cluster using the data set shown in Figure 3. The enrichment plot presents the gene sets of SMAD2 or SMAD3 target genes. **E** Z-score of Smad3 regulon activity by cell type and genome, analyzed by pySCENIC using the data set shown in Figure 3.

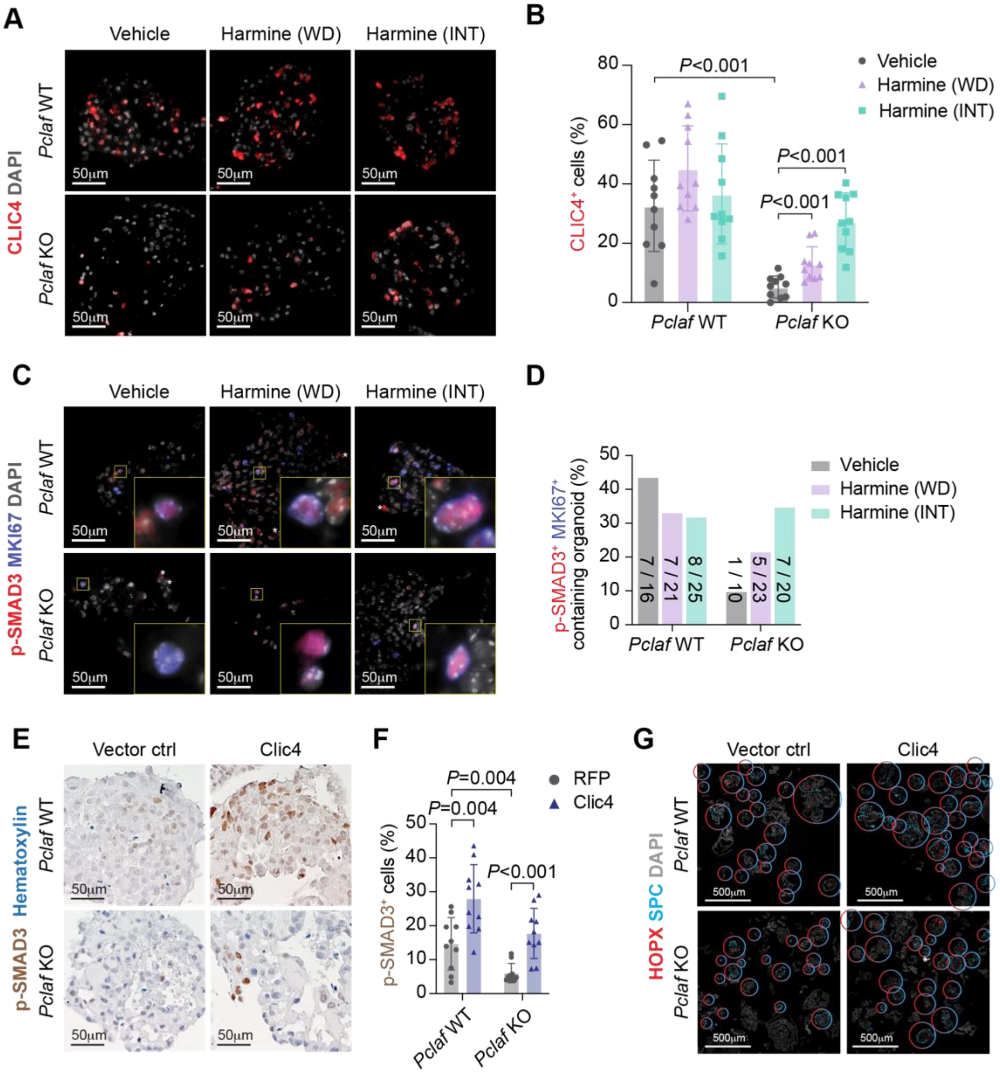

Supplementary Fig. 15. Pharmacological or genetic activation of the DREAM axis rescues *Pclaf* KO-inhibited TGF-β signaling.

A-**D** LOs treated with harmine (shown in Figure 4E) were immunostained with indicated antibodies. Representative images of IF staining for CLIC4 (**A**). Quantification of the CLIC4$^+$ cells (n=10) (**B**). Representative images of IF staining for p-SMAD3 and MKI67 (**C**). Quantification of LOs containing p-SMAD3 and MKI67 double-positive cells (n=10) (**D**). **E-G** Isolated lung epithelial cells were transduced with *RFP*- or *Clic4*-expressing lentiviruses and cultured with LO. Representative images of *Pclaf* WT and *Pclaf* KO LOs at day 14, immunostained for p-SMAD3 (**E**). Quantification graph of p-SMAD3$^+$ cells (n=10) (**F**). Images of IF staining for HOPX (AT1) and SPC (AT2) on day 14. The circle color indicates the relative ratio of HOPX$^+$ to SPC$^+$ cells (**G**). Two-tailed Student's *t*-test; error bars: SD. Representative images are shown (n≥3).

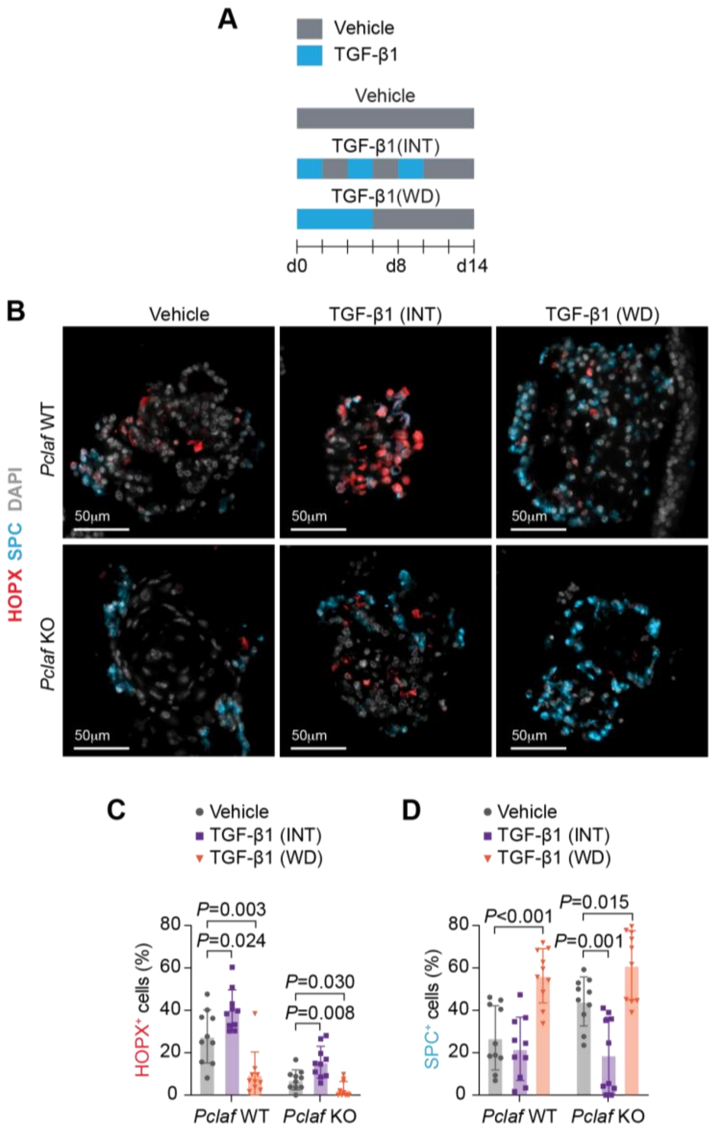

Supplementary Fig. 16. TGF-β rescues *Pclaf* KO-impaired cell plasticity.

Murine lung epithelial cells were cultured for LOs under stimuli of TGF-β1 (2 ng/ml). TGF-β1 was used to treat LOs for the first 6 days and withdrawn (WD). Alternatively, LOs were cultured with TGF-β1 intermittently (INT) at the indicated time points. **A** Experimental scheme for LO culture. **B** Representative images of IF staining for HOPX (AT1) and SPC (AT2) on day 14. **C**, **D** Quantification of $HOPX^+$ (n=10) (**C**) and SPC^+ cells (n=10) (**D**). Two-tailed Student's *t*-test; error bars: SD. The representative images are shown ($n \geq 3$). TGF-β1 (INT) rescued *Pclaf* KO-impaired alveolar cell plasticity (AT1 cell generation from AT2 cells). In contrast, TGF-β1 (WD) inhibited AT1 cell generation regardless of *Pclaf* WT and *Pclaf* KO. These data suggest that the temporal activity of TGF-β signaling is pivotal for AT2-to-AT1 cell transition. Of note, this experiment was designated by two rationales. First, signaling pathways elicit various outcomes depending on the spatiotemporal and dosage of signaling cues [2-4]. For example, intermittent or continuous stimulation of parathyroid hormone (PTH) can result in either increased or decreased bone mass, respectively [5]. Second, the balance between proliferation and differentiation is critical in organoid culture in general and TGF-β signaling has been shown to inhibit the proliferation of alveolar cells [6]. Since TGF-β generally inhibits cell proliferation, we thought that stimulation of TGF-β during the whole period would severely inhibit organoid formation and growth. Thus, to reduce the inhibitory effect of TGF-β, we cultured the LOs under TGF-β stimulation of half period of culture with two different models. The INT model aimed to continuously alter the organoid culture condition between TGF-β1 stimulation and depletion, while the WD model aimed to provide continuous TGF-β1 stimulation at the early stage of LO culture.

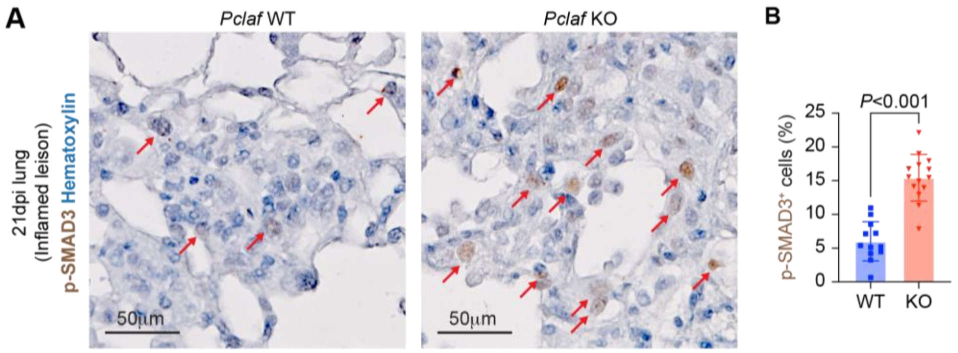

Supplementary Fig. 17. Elevated TGF-β signaling in inflamed lesions of *Pclaf* KO lung tissues

A Images of immunostaining for p-SMAD3 of the inflamed lesion from *Pclaf* WT (n=12) and *Pclaf* KO (n=14) lung tissues at 21 dpi. **B** Quantification graph of p-SMAD3$^+$ cells. Two-tailed Student's *t*-test; error bars: SD. Representative images are shown (n≥3).

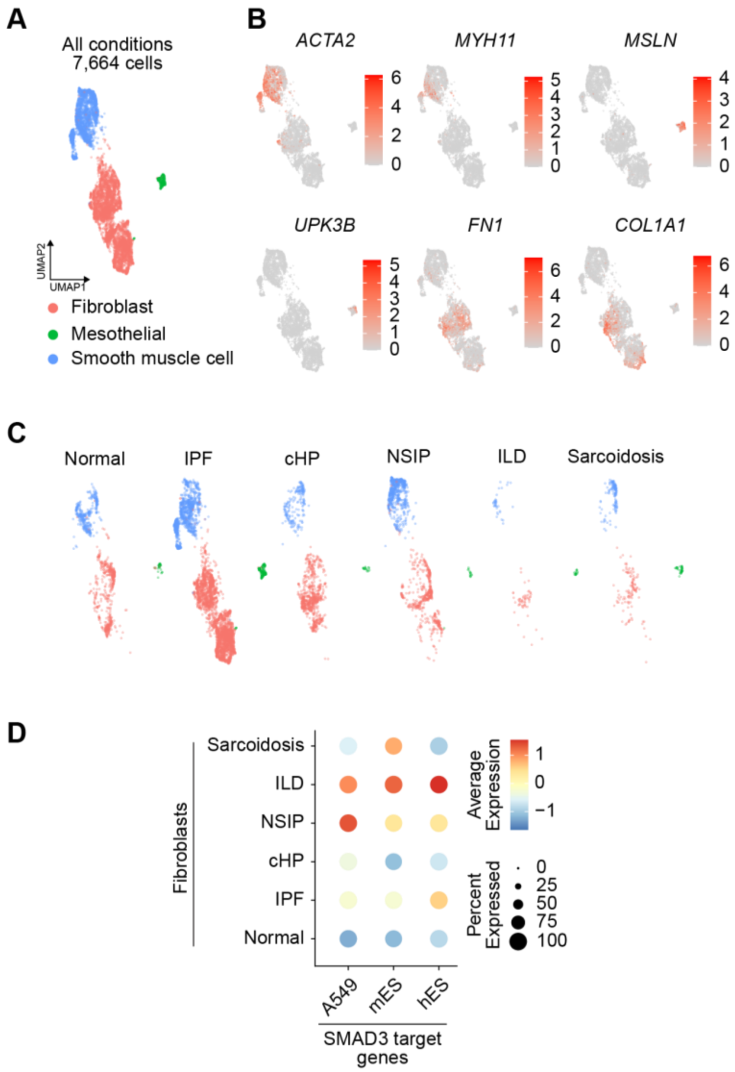

Supplementary Fig. 18. Elevated TGF-β signaling in lung fibroblasts of IPF patients
A UMAP showing the Mesenchymal compartment (*EPCAM*-, *PTPRC*-, and *PECAM*- cells) from the human scRNA-seq dataset (GSE135893; normal, IPF, cHP, NSIP, ILD, and Sarcoidosis). **B** Feature plots of indicated marker genes: *ACTA2* and *MYH11* for smooth muscle cells; *MSLN* and *UPK3B* for mesothelial cells; *FN1* and *COL1A1* for fibroblasts. **C** UMAP embedding displays cells colored by cell types split by each disease type. **D** Dot plots showing the expression of indicated genes and module scores of SMAD3-target gene sets in human lung fibroblasts.

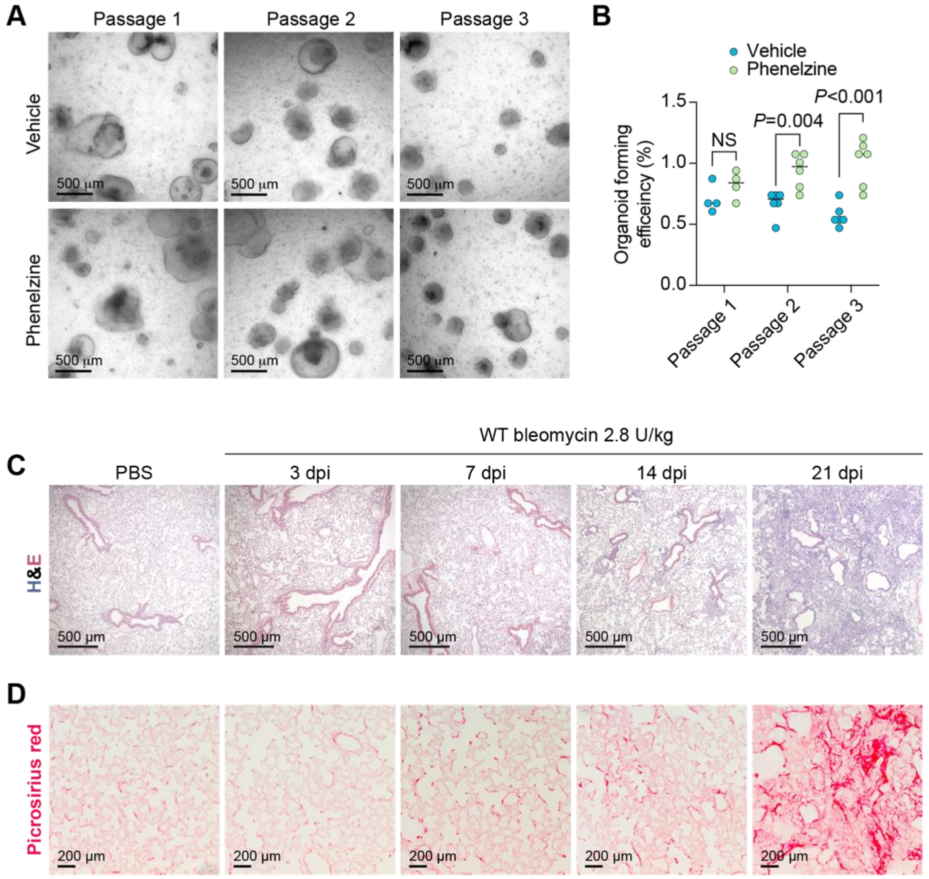

Supplementary Fig. 19. Phenelzine promotes LO formation.

A Images of LOs with phenelzine (10 μM) at 12 days of indicated passage. **B** Quantification graph of lung OFE at 12 days of passage (n=4 for passage 1, n=6 for passage 2 and 3). **C, D** 8-week-old C57BL/6 mice were treated with PBS (n=3) or bleomycin (2.8 U/kg, n=3 of each group) by intratracheal instillation. Lung tissues were collected at indicated time points. Lungs with PBS were collected at the same time point of 21 dpi. Representative images of H&E staining at the indicated time points (**C**). Representative images of picrosirius red staining at the indicated time points (**D**). Two-tailed Student's *t*-test; error bars: SD. Representative images are shown (n≥3).

Supplementary Fig. 2A

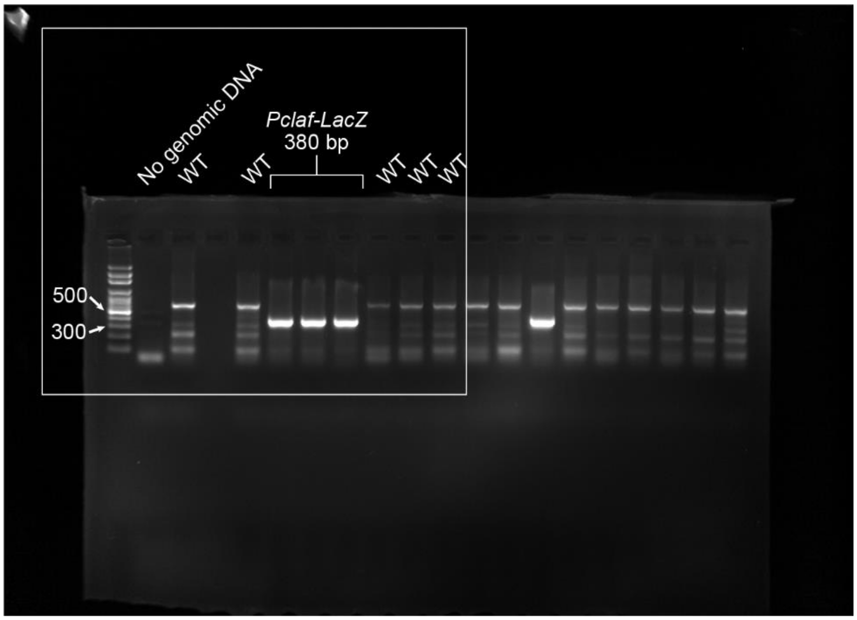

Supplementary Fig. 10B

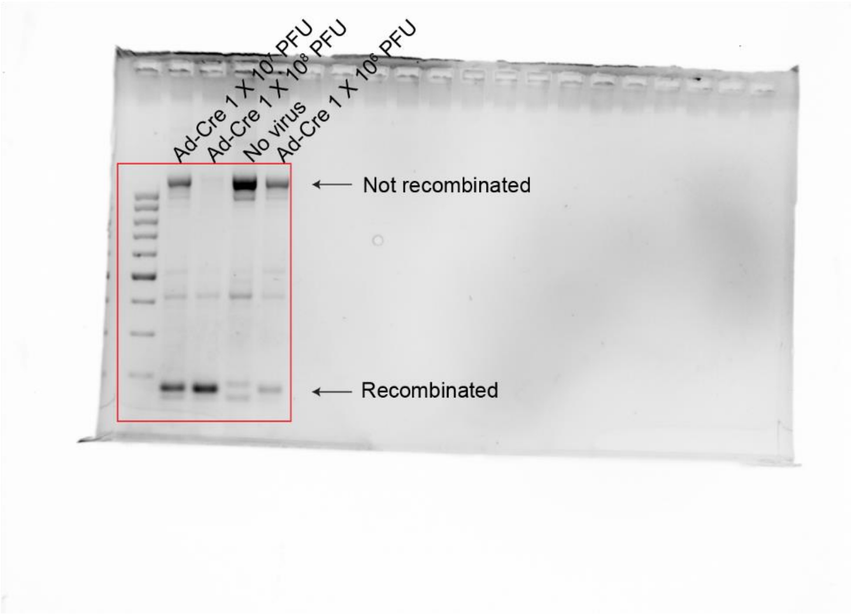

225 **Supplementary Fig. 20. Source data**
226 Raw uncropped agarose gel images shown in Supplementary Figure 2A and 10B.

Supplementary References

1 Kim, M. J. *et al.* PAF-Myc-Controlled Cell Stemness Is Required for Intestinal Regeneration and Tumorigenesis. *Dev. Cell* **44**, 582-+, doi:10.1016/j.devcel.2018.02.010 (2018).

2 Fowell, D. J. & Kim, M. The spatio-temporal control of effector T cell migration. *Nat Rev Immunol* **21**, 582-596, doi:10.1038/s41577-021-00507-0 (2021).

3 Araque, A. *et al.* Gliotransmitters travel in time and space. *Neuron* **81**, 728-739, doi:10.1016/j.neuron.2014.02.007 (2014).

4 Barrientos, S., Stojadinovic, O., Golinko, M. S., Brem, H. & Tomic-Canic, M. Growth factors and cytokines in wound healing. *Wound Repair Regen* **16**, 585-601, doi:10.1111/j.1524-475X.2008.00410.x (2008).

5 Silva, B. C. & Bilezikian, J. P. Parathyroid hormone: anabolic and catabolic actions on the skeleton. *Curr Opin Pharmacol* **22**, 41-50, doi:10.1016/j.coph.2015.03.005 (2015).

6 Riemondy, K. A. *et al.* Single cell RNA sequencing identifies TGFβ as a key regenerative cue following LPS-induced lung injury. *JCI Insight* **5**, doi:10.1172/jci.insight.123637 (2019).
