# Supplementary material for: PCLAF-DREAM drives alveolar cell plasticity for lung regeneration
Source: Nat Commun. 2024 Oct 24;15:9169. doi: 10.1038/s41467-024-53330-1 (PMC11502753; doi:10.1038/s41467-024-53330-1)
Supplement: Supplementary file 3 — Description of additional supplementary files [file 41467_2024_53330_MOESM3_ESM.pdf]

Description of Additional Supplementary Files

Supplementary Data 1. Marker genes of each cell type in mouse lung scRNA-seq data. The list of marker genes for each cell type identity of mouse lung scRNA-seq data (GSE1412259), analyzed by the 'FindAllMarkers' function of Seurat.

Supplementary Data 2. Marker genes of each cell type in human lung scRNA-seq. The list of marker genes for each cell type identity of human lung scRNA-seq data (GSE135893), analyzed by the 'FindAllMarkers' function of Seurat.

Supplementary Data 3. Marker genes of each cell type in mouse lung scRNA-seq from Pclaf WT and Pclaf KO mice at 7 dpi of bleomycin. The list of marker genes for each cell type identity of Pclaf WT and KO mouse lung epithelial cells (collected from mice at 7 dpi of bleomycin instillation), analyzed by 'FindAllMarkers' function of Seurat.

Supplementary Data 4. List of gene sets from of input into CLUE database. The list of gene sets of input into the CLUE database. Gene sets of the PAPCs (Pclaf WT vs. Pclaf KO scRNA-seq shown in Figure 2). Gene sets of KP cells (shPclaf vs. shControl [shCon]) and H1792 cells (shPCLAF vs. shControl [shCon]) were identified from the bulk RNA-seq data (GSE136571 and GSE147305, respectively).

Supplementary Data 5. List of drug candidates identified from the CLUE database. This spreadsheet has the output of the CLUE database with each gene sets. Each output was derived from the input gene sets listed in Supplementary Data 4; gene sets of PAPCs (Pclaf WT vs. Pclaf KO), KP cells (shPclaf vs. shControl [shCon]) and H1792 cells (shPCLAF vs. shControl [shCon]). CLUE outputs (drug candidates) with scores higher than 1.5 were selected.

Supplementary Data 6. Gene ontology of DREAM-target genes generated from the PANTHER database. The list of gene ontology (GO) (molecular function) of DREAM-target genes analyzed by the PANTHER db 7. The gene set of DREAM-target genes (968 genes; FISCHER_DREAM_TARGETS) was analyzed for the statistical overrepresentation test of GO molecular function complete.

Supplementary Data 7. Antibody and primer information. This spreadsheet has all information related to antibodies (manufacturers, catalog numbers, dilution rates, and antigen retrieval methods) and primers (sequences for cloning and mRNA quantification).

Supplementary Data 8. Gene sets used for module score analysis. The list of gene sets used for module score using with scRNA-seq dataset. List of genes in DREAM target genes, Sox9-based progenitor genes, MYC target genes, SMAD3 target genes from A549 cells, SMAD3 target genes from mouse embryonic cells, and SMAD3 target genes from human embryonic cells.
