# Supplementary material for: PCLAF-DREAM drives alveolar cell plasticity for lung regeneration
Source: Nat Commun. 2024 Oct 24;15:9169. doi: 10.1038/s41467-024-53330-1 (PMC11502753; doi:10.1038/s41467-024-53330-1)
Supplement: Supplementary file 12 — Reporting Summary [file 41467_2024_53330_MOESM12_ESM.pdf]

nature portfolio

Corresponding author(s): Bongjun Kim, Jae-Il Park

Last updated by author(s): Oct 2, 2024

Reporting Summary

Nature Portfolio wishes to improve the reproducibility of the work that we publish. This form provides structure for consistency and transparency in reporting. For further information on Nature Portfolio policies, see our Editorial Policies and the Editorial Policy Checklist.

Statistics

For all statistical analyses, confirm that the following items are present in the figure legend, table legend, main text, or Methods section.

n/a	Confirmed	
☐	☒	The exact sample size (*n*) for each experimental group/condition, given as a discrete number and unit of measurement
☐	☒	A statement on whether measurements were taken from distinct samples or whether the same sample was measured repeatedly
☐	☒	The statistical test(s) used AND whether they are one- or two-sided<br>*Only common tests should be described solely by name; describe more complex techniques in the Methods section.*
☒	☐	A description of all covariates tested
☐	☒	A description of any assumptions or corrections, such as tests of normality and adjustment for multiple comparisons
☐	☒	A full description of the statistical parameters including central tendency (e.g. means) or other basic estimates (e.g. regression coefficient) AND variation (e.g. standard deviation) or associated estimates of uncertainty (e.g. confidence intervals)
☒	☐	For null hypothesis testing, the test statistic (e.g. *F*, *t*, *r*) with confidence intervals, effect sizes, degrees of freedom and *P* value noted<br>*Give P values as exact values whenever suitable.*
☒	☐	For Bayesian analysis, information on the choice of priors and Markov chain Monte Carlo settings
☒	☐	For hierarchical and complex designs, identification of the appropriate level for tests and full reporting of outcomes
☐	☒	Estimates of effect sizes (e.g. Cohen's *d*, Pearson's *r*), indicating how they were calculated

Our web collection on statistics for biologists contains articles on many of the points above.

Software and code

Policy information about availability of computer code

Data collection	GSE141259; GSE135893
Data analysis	The code used to reproduce the analyses described in this manuscript can be accessed via Zonodo (https://zenodo.org/records/13871131) and will also be available upon request. Softwares and Packages used in this study were described below.<br><br>Softwares<br>R 4.0.3<br>Python 3.9.7<br><br>R packages<br>Seurat 4.1.0<br>CytoTRACE 0.3.3<br>scProportionTest 0.0.0.9000<br>fgsea 1.16.0<br>dplyr 1.0.8<br>ggplot2 3.3.5<br>matrix 1.4-0<br>ade4 1.7-18<br>presto 1.0.0<br>SeuratDisk 0.0.0.9019<br>SeuratWrappers 0.3.0

Nebulosa 1.0.2
Cowplot 1.1.1
dittoSeq 1.2.6
ggpubr 0.4.0
scClustViz 1.3.8

Python Packages
ScVelo 0.2.4
pyscenic 0.11.2
pandas 1.3.3
scanpy 1.8.1
numpy 1.21.2
scipty 1.7.1
Loompy 3.0.6
Anndata 0.7.6
umap 0.5.2
scikit-learn 1.0.2
statsmodels 0.13.1
python-igraph 0.9.9
pynndescent 0.5.6

For manuscripts utilizing custom algorithms or software that are central to the research but not yet described in published literature, software must be made available to editors and reviewers. We strongly encourage code deposition in a community repository (e.g. GitHub). See the Nature Portfolio guidelines for submitting code & software for further information.

Data

Policy information about availability of data

All manuscripts must include a data availability statement. This statement should provide the following information, where applicable:

- Accession codes, unique identifiers, or web links for publicly available datasets
- A description of any restrictions on data availability
- For clinical datasets or third party data, please ensure that the statement adheres to our policy

scRNA-seq data are available via the Gene Expression Omnibus (GSE205815; https://www.ncbi.nlm.nih.gov/geo/query/acc.cgi?acc=GSE205815).

Research involving human participants, their data, or biological material

Policy information about studies with human participants or human data. See also policy information about sex, gender (identity/presentation), and sexual orientation and race, ethnicity and racism.

Reporting on sex and gender	N/A
Reporting on race, ethnicity, or other socially relevant groupings	N/A
Population characteristics	N/A
Recruitment	N/A
Ethics oversight	N/A

Note that full information on the approval of the study protocol must also be provided in the manuscript.

Field-specific reporting

Please select the one below that is the best fit for your research. If you are not sure, read the appropriate sections before making your selection.

☒ Life sciences ☐ Behavioural & social sciences ☐ Ecological, evolutionary & environmental sciences

For a reference copy of the document with all sections, see nature.com/documents/nr-reporting-summary-flat.pdf

Life sciences study design

All studies must disclose on these points even when the disclosure is negative.

Sample size	For all in vitro experiment, the number of samples sizes are three or above. For animal studies, sample sizes were estimated by power calculation.

Data exclusions	No samples or animals were excluded. Also, the criteria were not pre-established in experiments.
Replication	All experiments were performed using at least three biological replicas unless specified. All experiments were successfully replicated.
Randomization	All mice were randomized in each groups.
Blinding	N/A

Reporting for specific materials, systems and methods

We require information from authors about some types of materials, experimental systems and methods used in many studies. Here, indicate whether each material, system or method listed is relevant to your study. If you are not sure if a list item applies to your research, read the appropriate section before selecting a response.

Materials & experimental systems

n/a	Involved in the study
☐	☒ Antibodies
☐	☒ Eukaryotic cell lines
☒	☐ Palaeontology and archaeology
☐	☒ Animals and other organisms
☒	☐ Clinical data
☒	☐ Dual use research of concern
☒	☐ Plants

Methods

n/a	Involved in the study
☒	☐ ChIP-seq
☒	☐ Flow cytometry
☒	☐ MRI-based neuroimaging

Antibodies

Antibodies used

IHC
anti-PCLAF (Abcam, ab226255, 1:100)
anti-PCLAF (Abcam, ab56773, 1:100)
anti-SMA (Abcam, ab5694, 1:200)
anti-RAGE (R&D, MAB1179-100, 1:100)
anti-SPC (Abcam, ab40879, 1:200)
anti-SCGB1A1 (Abcam, ab40873, 1:200)
anti-Ac-TUB (Sigma, T6793, 1:200)
anti-HOPX (Santacruz, sc-398703, 1:100)
anti-CLIC4 (Cell signaling, 12644S, 1:200)
anti-p-SMAD3 (S423 + S425) (Abcam, ab52903, 1:200)
anti-KI67 (Cell signaling, 9449S, 1:200)
anti-FLAG (Abcam, ab205606, 1:200)
anti-CC3 (Cell signaling, 9664s, 1:200)
anti-CDH1 (BD, 610182, 1:200)
anti-KRT8 (DSHB, DSHB TROMA-I, 1:200)
anti-LCN2 (R&D, AF1857-SP, 1:200)
anti-CD45 (Abcam, ab10558, 1:100)
anti-CD44 (BD, 550538, 1:200)
anti-FN (BD, 610078, 1:200)

ChIP
anti-FLAG (Sigma, F3040, 1:200)
anti-PCLAF (Abcam, ab226255, 1:200)

Validation

Allantibodieswerecommerciallyvalidated(seemanufacturer'swebsitelinkbelow)
anti-PCLAF (Abcam, ab226255)
https://www.abcam.com/products/primary-antibodies/kiaa0101-antibody-ab2anti-PCLAF (Abcam, ab56773,
anti-PCLAF (Abcam, ab56773, 1:100)
https://www.abcam.com/products/primary-antibodies/kiaa0101-antibody-ab56773.html
anti-SMA (Abcam, ab5694)
https://www.abcam.com/products/primary-antibodies/alpha-smooth-muscle-actin-antibody-ab5694.html
anti-RAGE (R&D, MAB1179-100)
https://www.rndsystems.com/products/mouse-rat-rage-antibody-175410_mab1179
anti-SPC (Abcam, ab40879)
https://www.abcam.com/products/primary-antibodies/prosurfactant-protein-c-antibody-ab40879.html
anti-SCGB1A1 (Abcam, ab40873)
https://www.abcam.com/products/primary-antibodies/uteroglobin-antibody-ab40873.html
anti-Ac-TUB (Sigma, T6793)
https://www.sigmaaldrich.com/US/en/product/sigma/t6793
anti-HOPX (Santacruz, sc-398703)

https://www.scbt.com/p/hop-antibody-e-1
anti-CLIC4 (Cell signaling, 12644S)
https://www.cellsignal.com/products/primary-antibodies/clic4-d2a7d-rabbit-mab/12644?_requestid=2136261
anti-p-SMAD3 (S423 + S425) (Abcam, ab52903)
https://www.abcam.com/products/primary-antibodies/smad3-phospho-s423--s425-antibody-ep823y-ab52903.html
anti-KI67 (Cell signaling, 9449S)
https://www.cellsignal.com/products/primary-antibodies/ki-67-8d5-mouse-mab/9449
anti-FLAG (Abcam, ab205606)
https://www.abcam.com/products/primary-antibodies/ddddk-tag-binds-to-flag-tag-sequence-antibody-epr20018-251-ab205606.html
anti-CC3 (Cell signaling, 9664s)
https://www.cellsignal.com/products/primary-antibodies/cleaved-caspase-3-asp175-5a1e-rabbit-mab/9664
anti-CDH1 (BD, 610182)
https://www.bdbiosciences.com/en-us/products/reagents/microscopy-imaging-reagents/immunofluorescence-reagents/purified-mouse-anti-e-cadherin.610182
anti-KRT8 (DSHB, DSHB TROMA-I)
https://dshb.biology.uiowa.edu/TROMA-I
anti-LCN2 (R&D, AF1857-SP)
https://www.rndsystems.com/products/mouse-lipocalin-2-ngal-antibody_af1857
anti-CD45 (Abcam, ab10558)
https://www.abcam.com/products/primary-antibodies/cd45-antibody-ab10558.html
anti-CD44 (BD, 550538)
https://www.bdbiosciences.com/en-us/products/reagents/flow-cytometry-reagents/research-reagents/single-color-antibodies-ruo/purified-rat-anti-mouse-cd44.550538
anti-FN (BD, 610078)
https://www.bdbiosciences.com/en-us/products/reagents/flow-cytometry-reagents/research-reagents/single-color-antibodies-ruo/purified-mouse-anti-fibronectin.610078
anti-FLAG (Sigma, F3040)
https://www.sigmaaldrich.com/US/en/product/sigma/f3040

Eukaryotic cell lines

Policy information about cell lines and Sex and Gender in Research

Cell line source(s)	H358 lung adenocarcinoma cell line was purchased from ATCC (ATCC, CRL5807).
Authentication	We have not performed additional authentication.
Mycoplasma contamination	Cells were negative for mycoplasma contamination
Commonly misidentified lines (See ICLAC register)	No commonly misidentified cell lines were used in this study

Animals and other research organisms

Policy information about studies involving animals; ARRIVE guidelines recommended for reporting animal research, and Sex and Gender in Research

Laboratory animals	Wild type C57BL/6J mice were purchased from Jackson laboratory (RRID: IMSR_JAX:000664).<br>Pclaf KO mice were generated in previous study. (2018. Developmental Cell. DOI: https://doi.org/10.1016/j.devcel.2018.02.010).<br>Pclaf-LacZ-neo mice were purchased from EMMA (EMMA ID: EM09820).<br>CMV-Cre mice were purchased from Jackson laboratory (IMSR_JAX:006054).<br>Rosa26-Flp1 mice were purchased from Jackson laboratory (RRID:IMSR_JAX:009086).<br>Sftpc-CreERT2; Rosa26-Sun1GFP mice were kindly provided by Dr. Jichao Chen (M.D. Anderson Cancer Center)<br>Pclaf-LacZ mice were established in this study by breeding Pclaf-LacZ-neo mice with CMV-Cre driver mice.<br>Pclaf-fl/fl mice were established in this study by breeding Pclaf-LacZ-neo mice with Rosa26-Flp1 driver mice.
Wild animals	The study did not involve wild animals
Reporting on sex	This information has not been collected, as it is irrelevant to our study.
Field-collected samples	The study did not involve field-collected samples.
Ethics oversight	All mouse experiments were approved by the MD Anderson Institutional Animal Care and Use Committee and performed under MD Anderson guidelines and the Association for Assessment and Accreditation of Laboratory Animal Care international standards.

Note that full information on the approval of the study protocol must also be provided in the manuscript.
